# Transferring functional annotations of membrane transporters on the basis of sequence similarity and sequence motifs

**DOI:** 10.1186/1471-2105-14-343

**Published:** 2013-11-28

**Authors:** Ahmad Barghash, Volkhard Helms

**Affiliations:** 1 Center for Bioinformatics, Saarland University, Postfach 15 11 50, 66041 Saarbrücken, Germany

**Keywords:** Membrane transporter, Functional classification, BLAST, HMMER, MEME, Substrate, TC classification system, Sequence homology

## Abstract

**Background:**

Membrane transporters catalyze the transport of small solute molecules across biological barriers such as lipid bilayer membranes. Experimental identification of the transported substrates is very tedious. Once a particular transport mechanism has been identified in one organism, it is thus highly desirable to transfer this information to related transporter sequences in different organisms based on bioinformatics evidence.

**Results:**

We present a thorough benchmark at which level of sequence identity membrane transporters from *Escherichia coli, Saccharomyces cerevisiae*, and *Arabidopsis thaliana* belong to the same families of the Transporter Classification (TC) system, and at what level these membrane transporters mediate the transport of the same substrate. We found that two membrane transporter sequences from different organisms that are aligned with normalized BLAST expectation value better than E-value 1e-8 are highly likely to belong to the same TC family (F-measure around 90%). Enriched sequence motifs identified by MEME at thresholds below 1e-12 support accurate classification into TC families for about two thirds of the sequences (F-measure 80% and higher). For the comparison of transported substrates, we focused on the four largest substrate classes of amino acids, sugars, metal ions, and phosphate. At similar identity thresholds, the nature of the transported substrates was more divergent (F-measure 40 - 75% at the same thresholds) than the TC family membership.

**Conclusions:**

We suggest an acceptable threshold of 1e-8 for BLAST and HMMER where at least three quarters of the sequences are classified according to the TC system with a reasonably high accuracy. Researchers who wish to apply these thresholds in their studies should multiply these thresholds by the size of the database they search against. Our findings should be useful to those who wish to transfer transporter functional annotations across species.

## Background

Prokaryotic and eukaryotic genomes each encode for hundreds of membrane transporter proteins that play essential roles for the cellular import and export of ions and small molecules. Furthermore, transporters mediate signal transduction processes catalyzing the export and uptake of signaling molecules. Therefore, the functional classification of membrane transporters is an important task. The available experimental knowledge about transporter function has been compiled in databases such as TCDB [1], TransportDB [2], SGD [3], and Aramemnon [4]. In these databases, the functional classification is normally done according to the hierarchical transporter classification (TC) system [5] adopted by the International Union of Biochemistry and Molecular Biology (IUBMB).

The TC system categorizes transporter sequences according to their class, subclass, (super) family, and subfamily on the basis of functional or phylogenetic information that is based on sequence similarity. An example for this classification would be the PTS Glucose-Glucoside (Glc) super family 4.A.1 that belongs to class ‘4’ group translocators and subclass ‘A’ phosphate transfer-driven group translocators. Subfamilies might correspond to transported substrates. A particular transporter sequence in such a family is identified by an extra digit to the right as e.g. 4.A.1.1.1.

A very important detail about each membrane transporter is of course the nature of its transported substrate molecule(s). As an alternative to the TC system, one may also classify transporters into different sets according to their substrates. It is presently unclear how such a substrate-based classification compares with the TC classification system. For example, the Aramemnon database lists members of five different TC families as phosphate transporters in *Arabidopsis thaliana*. In fact, many databases ignore the fourth digit (subfamily) of the TC system that normally refers to the main substrate. Schaadt and Helms have recently reported that membrane transporters from *Arabidopsis thaliana* that either transport amino acids, oligopeptides, phosphate, or sugar molecules can be distinguished from each other based on their amino acid composition [6,7].

An important research question for membrane biology is whether two membrane transporters in organisms X and Y that show a certain sequence similarity will have the same function or not. Previous computational work in this area classified transporters using sequence homology and motif searches [8,9], amino acid composition [10], and substrate specificity [6]. Interestingly, no study has so far critically analyzed the reliability margins of the individual features. In the general context of protein function, the Pfam repository of protein families has become a quasi-standard. Pfam employs so-called gathering thresholds that are manually curated, family-specific, bit score thresholds that are chosen by Pfam curators at the time a family is built. The threshold used recently corresponds roughly to ‘safe’ *E*-value thresholds of ~10^-2^[11]. In the TC system, the standard used for establishing homology between two proteins is 9 standard deviations (SDs). This corresponds to a probability of 10^-19^ that the degree of similarity observed arose by chance [12]. Chen and colleagues have recently assessed the performance of different orthology detection strategies for eukaryotic genomes [13].

Here, we have selected the three important model systems *Escherichia coli* (in the following abbreviated as *Ec*)*, Saccharomyces cerevisiae* (*Sc*), and *Arabidopsis thaliana* (*At*) that belong arguably to the best characterized species in terms of transport processes. Analyzing homolog databases we found that *Sc* and *At* have more homologs compared to pairs (*Sc*, *Ec*) and (*Ec*, *At*) what reflects the smaller phylogenetic distance between *Sc* and *At.* According to the InParanoid database [14], 7173 out of the 26207 *At* genes (27.4%) have homologs in *Sc* and 2921 out of the 5884 *Sc* genes (49.6%) have homologs in *At*. For comparison, 933 *Sc* genes (15.8%) have homologs in *Ec* and 822 out of 4149 *Ec* genes (19.8%) have homologs in *Sc*. Finally, only 2778 *At* genes (10.6%) have homologs in *Ec* and 1168 *Ec* genes (28.1%) have homologs in *At*. Along the same lines, the *Arabidopsis* sequencing project revealed that a much higher percentage of the proteins in the 12 major functional subsets of the *At* genome had a BLASTP match with E < 10^-30^ to a protein from *Sc* (17–50)% than to a protein from *Ec* (5–32)% [15].

We used three different approaches to transfer transporter functional annotation between the three organisms by relating the level of sequence identity to the functional similarity between the three studied organisms. In this study, we will term this comparison “functional classification”. For this, we used the approaches BLAST that generates alignments that optimize a measure of local similarity [16], HMMER that searches for sequence homologs and performs protein sequence alignment using probabilistic methods [17], and MEME that performs motif discovery in protein sequences on the basis of expectation maximization [18]. So far there seem to be no accepted fixed thresholds for the prediction scores of the three tools. Therefore, different studies tend to use their own suitable set of thresholds [11-13,19-21]. Our study establishes a set of thresholds under which the transporter function can safely be transferred between the three model organisms.

## Results and discussion

**Figure 1:**
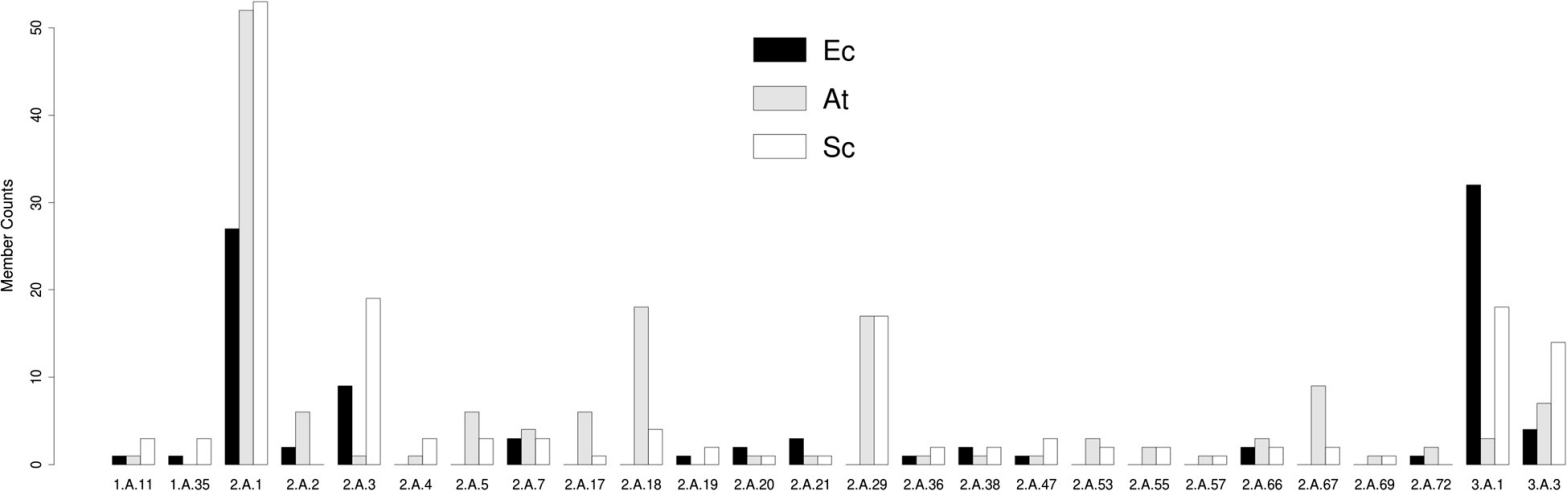
**Distribution of transporters among the TC families.** Common *Ec, At,* and *Sc* TC families with member counts. Most families belong to the Electrochemical Potential Driven Transporters (class 2) and the Primary Active Transporters TC classes (class 3). Shared TC families in the searched organism with more than 2 members were used for MEME motif analysis.

In this work, we perform functional classification of transporter TC families and of transported substrate molecule using datasets from three model organisms. Our aim is to provide a simple guideline to biologists who wish to get a quick information whether available functional information about a transporter in species X may be transferred to another transporter sequence identified e.g. by BLAST search in species Y. Table 1 provides an overview over the main data sets used in this work. Figure 1 lists common TC families between the three organisms and the distribution of transporters among them. Additional file 1: Tables S1-S3 list all used transporters in this study, their TC families, substrates, and their Pfam description.

**Table 1:** Datasets

	***Ec***	***At***	***Sc***
**Number of transporters with TC family annotated**	156	158	177
**Number of transporters with substrate annotation**	155	158	848
**Number of transporters with TC family and substrate annotation**	155	158	177
**Metal transporters**	10	13	22
**Phosphate transporters**	5	19	6
**Sugar transporters**	27	47	24
**Amino acid transporters**	30	16	27

Membrane transporters with experimental annotation downloaded from TransportDB, Aramemnon and SGD for *Ec, At* and *Sc*, respectively. Only transporters with annotated TC and substrate families were considered in this work.

**Figure 2:**
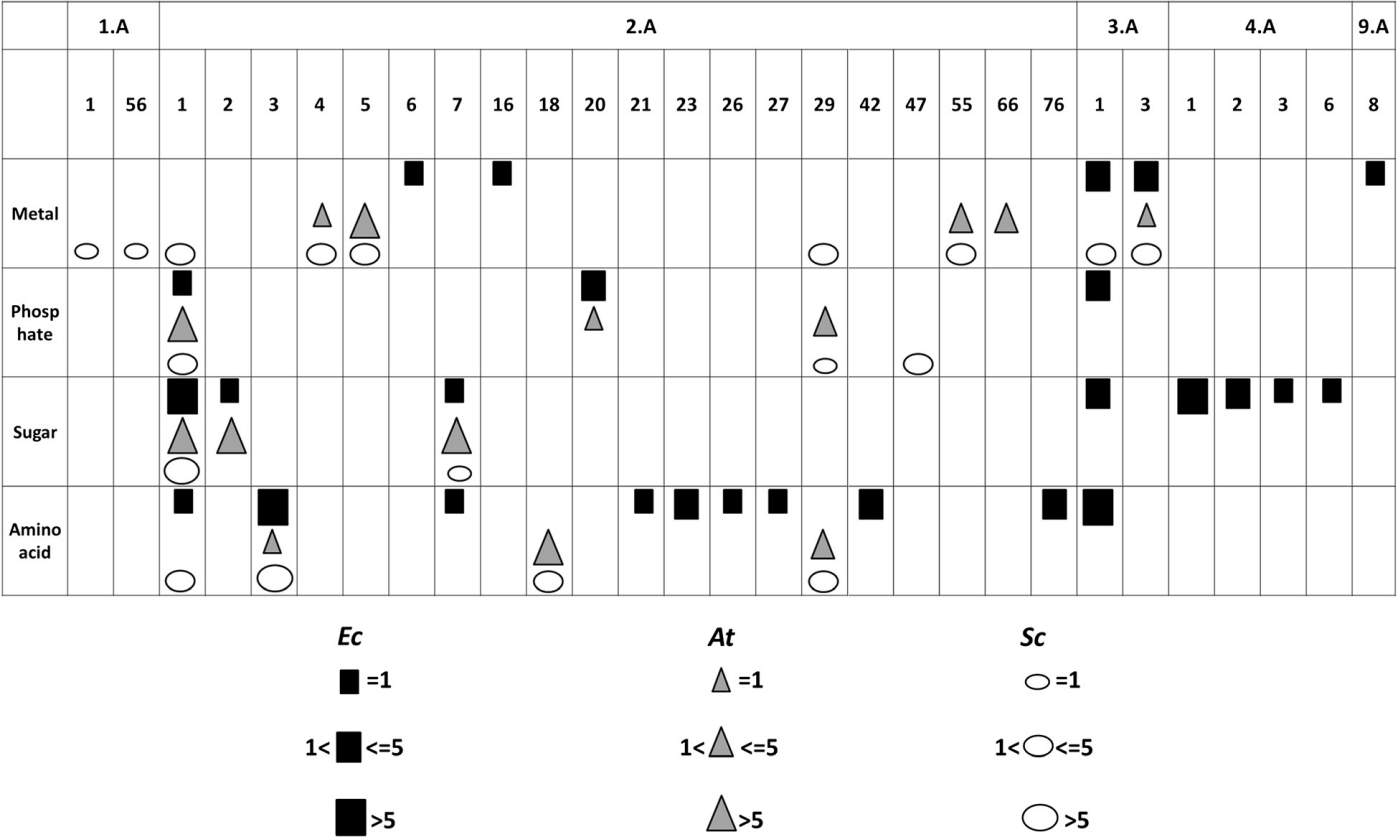
**Distribution of transporters from the four selected substrate families among the different TC families.** Distribution of metal, phosphate, sugar, and amino acid transporters among the different TC families in the three organisms; *Ec* (squares), *At* (triangles) and *Sc* (ovals). The size of the symbols indicates the number of members of this class. See Additional file 1: Tables S1-S3 for more details.

Beside the TC analysis, we also created substrate families of transporters that are annotated to transport the same substrate. For each organism, we collected four large groups of transporters that have been experimentally shown to catalyze the transport of either metal ions, phosphates, sugars, or amino acids. Metal ion transporters account for about 25% of the complete substrate dataset in each organism. *Sc* contains twice as many metal ion transporters as *Ec* and *At*[22]. This can possibly be related with the existence of metallothionein proteins in yeast that function as a metal storage [23]. *At* contains three times as many phosphate transporters as *Ec* and four times as many as in *Sc*. This is probably due to the essential role of phosphate regulating the *At* root system [24-26]. Sugar transporters in *At* even account for 50% of the complete substrate dataset which is twice as many as in *Ec* and *Sc*. One possible explanation for this is that plants need sugar to complete photosynthesis [27]. *Ec* and *Sc* contain twice as many amino acid transporters as *At*. Figure 2 provides an overview to which TC families the members of the created substrate families belong. We noticed that the transporters for these four substrates are spread over many different TC families.

## Matching TC families

In this work, we used BLAST for aligning all transporter sequences of one organism against their TC analogues in the two other organisms. Then, we calculated the accuracy measures precision, recall, and F-measure (eq. 1–3) for various E-value thresholds. BLAST multiplies the significance of a hit by the total number of residues in the database. Thus, to make the obtained results independent from the size of the searched database we divided the E-values by the size of the DB that we were BLASTing against. In this way E-values from searches against different TC sets or substrates sets are comparable to each other. In the following, we will term the normalized BLAST results "normalized E-values". As an example, we BLASTed *Sc* transporter YDR342C either against the *At* dataset (23,567 residues) or against the non-redundant (nr) database of 2011 with 3,877,139,759 residues. Among the matching sequences, we identified the Arabidopsis transporter At3g19940 in both BLAST runs with an E-value of 1e-58 when searching against the *At* dataset and 1e-53 when matching against nr. This difference of reported E-values matches the ratio of the database sizes.

On the other hand, when computing the accuracy measures, we multiplied the results by the member count of each family and then averaged over all TC families considered in order to account for the different member count of each family, see Table 2. The last row shows the percentage of transporters that remained unclassified at the given threshold. These are transporters from one organism belonging to the shared TC families that do not share sequence identity better than the given E-value to any transporter in the shared TC family from the other organism.

**Table 2:** BLAST sequence homology search results within TC families in the three organisms

	***Ec *– *At***					***Ec *– *Sc***					***Sc *– *At***				
	**1e-20**	**1e-16**	**1e-12**	**1e-8**	**1e-4**	**1e-20**	**1e-16**	**1e-12**	**1e-8**	**1e-4**	**1e-20**	**1e-16**	**1e-12**	**1e-8**	**1e-4**
**Precision [%]**	83.3	84.4	86.7	90.0	60.3	78.6	79.6	79.6	87.8	64.1	84.7	85.4	97.5	97.5	54.1
**Recall [%]**	83.3	84.4	86.7	90.0	76.2	78.6	79.6	79.6	87.8	65.1	84.7	85.4	97.5	97.5	62.9
**F-measure[%]**	83.3	84.4	86.7	90.0	64.7	78.6	79.6	79.6	87.8	63.6	84.7	85.4	97.5	97.5	55.2
**Unclassified [%]**	82.2	52.2	37.8	25.6	0.0	56.1	44.9	40.8	29.6	0.0	48.4	43.3	35.0	19.1	0.0

Accuracy measures of the BLAST prediction results for finding homologous transporter pairs in the *Ec*-*At*, *Ec*-*Sc*, and *Sc*-*At* comparison that belong to the same TC family for various E-value thresholds. The results were normalized by the size of the reference database (see text). Both precision and recall have a peak at thresholds 1e-12 or 1e-8 but showed lower accuracies under other thresholds. The unclassified percentage decreases as the thresholds’ values increase.

At the strictest threshold of 1e-20, the assignment of TC family has very high confidence but more than 80% of the sequences cannot be assigned for the *Ec*-*At* comparison and about half in the *Ec*-*Sc* and *Sc*-*At* comparisons. When the threshold is made more permissive, the number of correct predictions increased with few false predictions. We found that the precision and recall increased until 1e-8 but at threshold 1e-4 the number of false predictions increased. As expected, the unclassified percentage decreased as the thresholds were made more permissive. Based on this comparison, a rather permissive normalized BLAST threshold of 1e-8 is very acceptable but 1e-4 can still be considered with caution. When using the absolute identity scores of the alignment instead of the extracted E-values, the results were untrustworthy. The TC family prediction of *Ec* transporters based on *Sc* transporters annotated more sequences than the prediction based on *At* transporters at the strictest thresholds. Additionally, the *Sc*-*At* analysis resulted in a higher accuracy compared to the *Ec*-*At* analysis.

We then applied HMMER to the same datasets as for BLAST and calculated the accuracy measures and the unclassified percentage in the same way. Table 3 shows the results obtained with HMMER. For the purpose of normalization, the results were divided by the number of found hits in the database that was searched against. Overall, the results are similar to those obtained with BLAST. However, HMMER results are slightly more accurate at loose thresholds and cover a wider annotation fraction at the strictest thresholds with few more false positives. The number of correctly predicted TC family members at the medium-strong thresholds of 1e-16 and 1e-8 is always equal or higher than with BLAST. HMMER also missed fewer points (false negatives) compared to BLAST. This is clearly reflected by the higher recall value calculated most of the times. It should be re-emphasized that the E-values are computed by the three programs used here in different ways and are, thus, not directly comparable. Also, we have applied different normalization procedures - as suggested by the developers - to normalize the results to per-residue or per-sequence levels.

**Table 3:** HMMER results for homology between TC families from the three organisms

	***Ec *– *At***					***Ec *– *Sc***					***Sc *– *At***				
	**1e-20**	**1e-16**	**1e-12**	**1e-8**	**1e-4**	**1e-20**	**1e-16**	**1e-12**	**1e-8**	**1e-4**	**1e-20**	**1e-16**	**1e-12**	**1e-8**	**1e-4**
**Precision [%]**	73.3	85.6	86.7	90.0	90.8	78.6	78.6	81.6	86.7	92.9	84.7	85.4	85.4	97.5	93.7
**Recall [%]**	73.3	85.6	86.7	90.0	92.1	78.6	78.6	81.6	86.7	92.9	84.7	85.4	85.4	97.5	96.0
**F-measure[%]**	73.3	85.6	86.7	90.0	91.4	78.6	78.6	81.6	86.7	92.9	84.7	85.4	85.4	97.5	94.7
**Unclassified [%]**	76.7	52.2	40.0	33.3	17.8	21.4	21.4	18.4	13.3	7.1	15.3	14.6	14.6	2.5	2.5

HMMER prediction results (sequence E-values) under the given E-value confidence thresholds. The results were normalized by the size of the reference database (see text). HMMER gave a better accuracy under loose thresholds compared to BLAST.

The decisions by HMMER appear similar to BLAST between the three organisms. Apparently, HMMER attained slightly higher precision for almost all thresholds compared to BLAST especially at loose thresholds. Additionally, in the *Ec*-Sc and the *Sc*-*At* analysis, HMMER made predictions for a larger fraction of the test set with a noticeably higher recall for thresholds till 1e-8 compared to BLAST. For threshold 1e-4, HMMER predicted a slightly smaller fraction of the test set compared to BLAST but HMMER reported much higher prediction accuracy. Hence, we suggest an acceptable HMMER threshold of 1e-4.

The enriched sequence motifs identified by MEME in sequences from one organism were subsequently searched in test sets of sequences from the other two organisms using the MAST program [28] from the MEME suite. Table 4 illustrates the results based on using motif searches for family classification of transporters. As can be expected, motif based searches performed better in families with many members such as 2.A.1. For loose thresholds, motif based classification showed lower precision compared to HMMER and BLAST but a comparable precision at the strictest thresholds of 1e-20 and 1e-16 as in *Ec*-*Sc* and *Sc*-*At* analysis. We suggest that motif based methods may be used beneficially in combination with other methods to support transporter classification. At looser thresholds than 1e-8, motif-based searches seem to lead to unreliable results and should be used with high caution.

**Table 4:** MAST results for the existence of predicted MEME motifs in TC families

	***Ec *– *At***					***Ec *– *Sc***					***Sc *– *At***				
	**1e-20**	**1e-16**	**1e-12**	**1e-8**	**1e-4**	**1e-20**	**1e-16**	**1e-12**	**1e-8**	**1e-4**	**1e-20**	**1e-16**	**1e-12**	**1e-8**	**1e-4**
**Precision [%]**	45.1	90.1	90.1	68.7	15.8	83.9	83.9	79.1	33.5	13.2	94.2	99.2	100.0	57.3	9.6
**Recall [%]**	45.1	90.1	90.1	89.1	51.9	83.9	83.9	83.9	65.3	25.8	94.2	99.2	100.0	79.3	36.6
**F-measure[%]**	45.1	90.1	90.1	76.4	21.6	83.9	83.9	81.3	42.6	16.2	94.2	99.2	100.0	64.3	13.0
**Unclassified [%]**	87.3	80.3	45.1	4.2	0.0	47.1	46.0	34.5	1.1	0.0	51.7	40.0	28.3	4.2	0.0

MAST results searching for motifs predicted by MEME in the *Sc* and *At* test sets. Despite the fact that all sequences were classified, prediction accuracy is generally low at loose thresholds and at the strictest threshold in the *Ec-At* analysis.

## Matching substrates families

In a second step, we used the same three methods to test whether annotations about the transported substrate can be transferred from one organism to the other. For this, we created four subsets of metal ions transporters, phosphate transporters, sugar transporters, and amino acid transporters. These are the four largest known substrate families and comprised 72 *Ec* transporters, 95 *At* transporters, and 79 *Sc* transporters, see Table 1.

As shown in Table 5, the results were markedly different from the TC family results. Despite the fact that BLAST reported acceptable prediction precision in the *Ec*-*At* and the *Sc*-*At* analysis, the program missed classification of many transporters. We noticed that sequences tend to match sequences from their TC families in other substrate families, rather than their analogues in the same substrate family. Thus, the precision for substrate classification is generally lower than for the TC classification, in particular for the *Ec*-*Sc* comparison. For instance, the metal transporter (YMR301C) from *Sc* was falsely matched to about one third of all *Ec* transporters in the four substrate families irrespective of their substrates since they belong to the same TC family (3.A.1).

**Table 5:** Homology search results within the four substrate families based on BLAST

	***Ec *– *At***					***Ec *– *Sc***					***Sc *– *At***				
	**1e-20**	**1e-16**	**1e-12**	**1e-8**	**1e-4**	**1e-20**	**1e-16**	**1e-12**	**1e-8**	**1e-4**	**1e-20**	**1e-16**	**1e-12**	**1e-8**	**1e-4**
**Precision [%]**	71.6	72.9	66.1	56.8	37.8	57.7	44.1	38.5	39.3	34.9	95.5	79.8	69.9	62.2	37.0
**Recall [%]**	93.1	93.1	93.8	90.8	55.6	85.2	84.6	82.7	75.6	51.5	100.0	100.0	100.0	100.0	100.0
**F-measure[%]**	78.9	80.5	71.5	61.3	42.3	64.3	50.6	43.2	43.6	35.7	97.2	87.0	79.0	73.6	52.1
**Unclassified [%]**	90.3	86.1	79.2	72.2	8.3	65.3	56.9	52.8	51.4	1.4	45.7	44.3	37.1	27.1	1.4

BLAST prediction results for the four created substrate families of metal ion, phosphate, sugar and amino acid transporters. The results were normalized by the size of the reference database (see text). Unlike the TC family prediction, a smaller fraction of transporters was correctly classified and many were misclassified.

Table 6 presents the HMMER prediction results for substrate families from the three organisms. Compared to BLAST, HMMER reported higher prediction accuracy in the *Ec*-*Sc* analysis but slightly lower prediction accuracy in *Ec*-*At* analysis at the strict thresholds such as in the TC comparisons. In fact, BLAST classified a slightly larger fraction of the test sets than HMMER in almost all runs. HMMER was also affected by transporters tending to match their TC family members in other substrate families rather than their homologues in the same substrate families.

**Table 6:** HMMER search results within the four chosen substrate families

	***Ec *– *At***					***Ec *– *Sc***					***Sc *– *At***				
	**1e-20**	**1e-16**	**1e-12**	**1e-8**	**1e-4**	**1e-20**	**1e-16**	**1e-12**	**1e-8**	**1e-4**	**1e-20**	**1e-16**	**1e-12**	**1e-8**	**1e-4**
**Precision [%]**	51.4	58.3	69.1	66.0	57.7	85.2	77.0	72.7	71.3	70.3	99.3	90.4	76.3	71.7	59.9
**Recall [%]**	51.4	58.3	100.0	93.5	88.7	83.8	82.4	82.3	78.4	74.6	96.2	95.3	93.4	90.1	86.0
**F-measure[%]**	51.4	58.3	75.9	70.3	61.9	81.9	75.5	73.3	71.1	69.0	97.2	91.4	78.6	75.4	68.2
**Unclassified [%]**	93.1	88.9	83.3	79.2	65.3	69.4	61.1	55.6	51.4	47.2	45.7	44.3	41.4	34.3	17.1

HMMER prediction results for substrate families. The results were normalized by the size of the reference database (see text). HMMER gave a slightly higher prediction accuracy than BLAST in the *Ec*-*Sc* analysis.

Table 7 shows MAST search results for MEME motifs from different substrate families. MEME gave weak predictions in all runs but in the *Sc*-*At* analysis. However, recall in the medium strict thresholds 1e-16 and 1e-8 in the *Ec*-*Sc* analysis is generally acceptable but accompanied with many misclassifications. In the *Ec*-*At* analysis the prediction accuracy was generally low. Here, even the strict threshold of 1e-20 is unreliable because it gave wrong assignments of substrates in two out of three analyses.

**Table 7:** MAST results for the existence of predicted MEME motifs in substrate families

	***Ec *– *At***					***Ec *– *Sc***					***Sc *– *At***				
	**1e-20**	**1e-16**	**1e-12**	**1e-8**	**1e-4**	**1e-20**	**1e-16**	**1e-12**	**1e-8**	**1e-4**	**1e-20**	**1e-16**	**1e-12**	**1e-8**	**1e-4**
**Precision [%]**	37.5	37.5	52.8	39.8	25.0	34.7	56.3	52.2	34.9	25.1	82.9	81.5	49.4	30.2	25.0
**Recall [%]**	37.5	37.5	73.2	48.7	44.2	41.7	81.5	85.5	50.4	40.3	96.7	93.0	79.7	39.3	31.7
**F-measure[%]**	37.5	37.5	61.3	43.8	30.1	37.9	58.5	60.2	40.9	29.4	87.7	85.9	58.8	30.3	27.3
**Unclassified [%]**	95.8	94.4	80.6	9.7	0.0	90.3	75.0	59.7	9.7	0.0	68.7	55.3	52.0	0.0	0.0

MAST results searching for up to 3 motifs predicted by MEME in each substrate family from *Sc* and *At*. Most members of the substrate families were correctly classified for threshold (1e-4) but only with a very low accuracy.

Surprisingly, 22 *Sc* sugar transporters were correctly classified from 3 motifs predicted by MEME in the *At* sugar substrate family. To the best of our knowledge, none of the three motifs have been annotated so far in databases such as [29]. Table 8 lists the regular expressions of these three motifs. The motifs were found around positions 420, 150, and 300 of the protein sequences, respectively.

**Table 8:** Enriched sequence motifs in At sugar transporters

**Approximate position**	**Regular expressions**
420	F[AS][WI][GS][WM]GP[LVI][GP]W[LVI][VI]PSEIFPL[ER][IL]R[SGA]A[GA][QG][SA][IL]A[VA][SAL]VN[WM][IFV]F[TNS]F[IL][IV][AGT]Q[SAT]FLS[ML]L[CE][AH]
150	F[LFI]IG[AS][LI][LV][MN][AG]FAPNVA[MV]LI[IV]GR[LI]L[LA]G[FI]G[VI]G[FL][AG][NS][QM]A[VA]P[VL]Y[IL][SA]E[IM][AS]PAKIRG[AG]
300	[GA][VI]G[LI][QP]F[FL]QQ[LF][TS]GIN[AV][VI][ML][FY]Y[AS]P[VT][IL]F[QK][TK]AGF

Regular expressions of the three motifs predicted in *At* sugar transporters that lead to correct predictions of 22 *Sc* sugar transporters at the second-strictest threshold of 1e-16.

### Application of established thresholds to human datasets

Next, we tested these thresholds on four *Hs* datasets. In comparison to the three model organisms, these datasets are likely much less complete. We used the three tools to align the *Hs* transporters using a set of transporters from *At* and *Sc* and to align *Ec* transporters using *Hs* transporters. The results are in line with the comparisons of the three model organisms. When using BLAST and HMMER, only a small fraction was annotated at strict thresholds but more were classified at more permissive thresholds. Using HMMER, about 50% of the transporters remain not annotated even at the loosest threshold of 1e-4 whereas using BLAST many more were annotated but with a very low prediction accuracy. The reason is that the *Hs* phosphate and metal transporters were not annotated using the *At* and *Sc* sets and even did not help in annotating the *Ec* transporters. However, sugar and amino acid transporters were mostly correctly annotated. Most annotations of *Hs* transporters were based on matching (*Hs*, *Sc*) pairs. In motif searches, two thirds of the *Hs* transporters were annotated at the threshold of 1e-16 but none were annotated at the strictest threshold of 1e-20, see Table 9. The complete results of matching (*Hs*, *At*) and (*Ec*, *Hs*) are listed in Additional file 2: Table S4.

**Table 9:** **Results of the three tools matching *Hs *** **and *Sc *** **sequences**

	**BLAST**					**HMMER**					**MEME**				
	**1e-20**	**1e-16**	**1e-12**	**1e-8**	**1e-4**	**1e-20**	**1e-16**	**1e-12**	**1e-8**	**1e-4**	**1e-20**	**1e-16**	**1e-12**	**1e-8**	**1e-4**
Precision	66.7	66.7	62.1	59.2	38.6	66.7	66.7	66.7	66.5	65.1	0.0	66.7	66.7	37.6	25.0
Recall	66.7	66.7	66.7	66.7	55.4	66.7	66.7	66.7	66.7	64.3	0.0	66.7	66.7	31.3	34.1
F-measure	66.7	66.7	64.1	62.1	45.1	66.7	66.7	66.7	66.6	64.7	0.0	66.7	66.7	34.1	27.4
Unclassified	66.7	60.0	60.0	60.0	6.7	66.7	66.7	60.0	60.0	53.3	100.0	33.3	33.3	0.0	0.0

*Hs* transporters were better annotated using *Sc* transporters compared to *At.* The results were corrected for the size of the reference database (see text). About half of the transporters remained unannotated in the HMMER runs. Two thirds of the human transporters were annotated using MEME at the threshold of 1e-16.

Additionally, we studied the pairwise global similarity of all organism pairs using the program ggsearch from the FASTA program suite. The results were generally similar to BLAST and HMMER results with a slightly lower accuracy at the loose thresholds and even lower accuracy at the stricter thresholds. Results are listed in Additional file 3: Tables S5-S6.

### Prediction of TC families in substrate families

**Figure 3:**
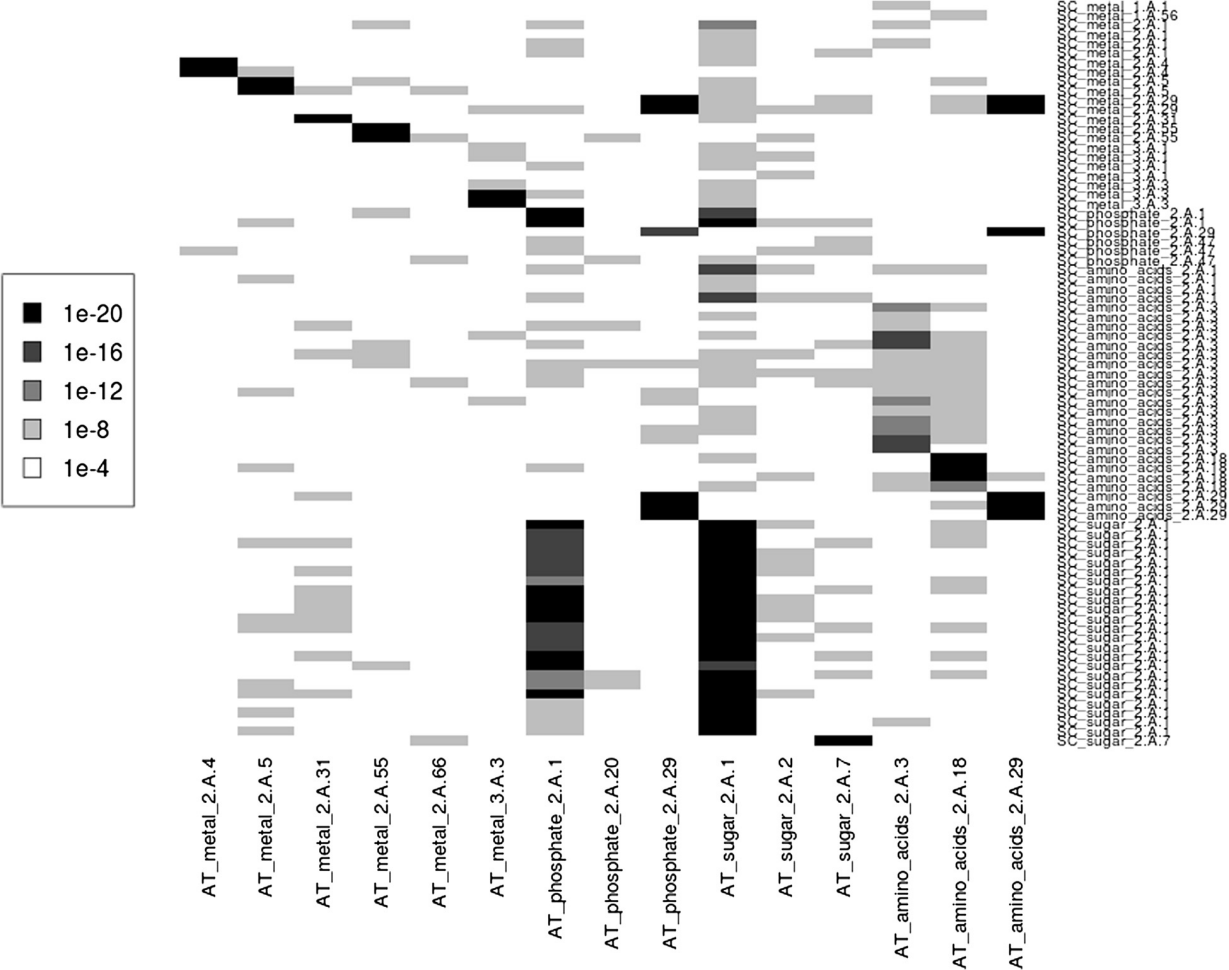
**Heatmap of BLASTing *Sc *substrate_TC families against *At *families.** BLAST homology search of 69 *Sc* transporters against 84 *At* transporters from 4 substrate families (amino acids, sugars, phosphates, metals) and 13 TC families (*Sc*) and 12 TC families (*At*). The grey scale follows a logarithmic scheme where white means no match better than normalized E < e-04 and black means the best matches better than E < e-20. Families generally match their substrate_TC families. However, they may also match TC families from different substrate_TC families.

Comparison of the two preceding sections shows that substrate families have less sequence similarity on average compared to TC families. Now, we tested the combination of both properties, see Figure 3. We performed this comparison in a systematic way. For this, we named the extracted families in the form “substrate family_TC family”. The four substrate families (amino acids, sugars, phosphates, metals) belong to 19 TC families in *Ec*, 13 in *At* and 14 in *Sc*. 7 families substrate-TC are shared between *Ec* and *At*, 7 also are shared between *Ec* and *Sc* and 11 are shared between *Sc* and *At*. Some TC families belong to many different substrate families like the family 3.A.1 that contains members of 4 *Ec* substrate families. We used BLAST to analyze the affiliation of test sequences toward their TC or substrate families. Here, only the best match of each substrate_TC family is considered. The heatmap in Figure 3 shows the tendency of *Sc* sequences to match their analogues from *At* TC or substrate families. Some *Sc* transporters matched strongly (black rectangles) their actual substrate_TC families from *At* like sugar_2.A.1, phosphate_2.A.1 and metal_2.A.55. However, most sequences from shared TC families had weaker matches to their TC families rather than their substrate families. Similar results were obtained in the *Ec-At* and *Ec-Sc* comparison, see Additional files 4: Figure S4 and Additional file 5: Figure S5. Thus, we suggest that it is beneficial to apply substrate information as a pre-filter for transporter TC family classification. On the other hand, transporters that transport the same substrate but belong to different TC families generally do not share noticeable sequence similarity. TC information can be the stand alone feature used to classify transporters but a little tuning by substrate information elevates the prediction accuracy. Misclassification will occur in the small substrate_TC families not in the big TC families.

## Limitations and implications

In some way, our analysis presented here is a bit “circular” since we employ tools to identify sequence pairs belonging to the same TC categories while the TC classification itself was established in part based on phylogenetic analysis that is again based on sequence similarity. However, in a practical use case it is far simpler to run a BLAST or FASTA analysis than to establish a complicated phylogeny. Hence, our results reflect to what extent simple sequence similarity captures the structure of the more elaborate TC classification.

When comparing the results of the four methods (BLAST, FASTA, HMMER3, MEME), the reader should not forget that different strategies are employed by each of the methods to derive E-values for the reported results. Hence, the results of different methods are not directly comparable.

Note that datasets to be used for motif discovery are typically cleaned up for sequence redundancy e.g. using BLASTCLUST with a 25% sequence identity threshold [30]. Here, we did not do this because this would significantly decrease the number of families in the TC dataset that can be used for analysis. Hence, the MEME analysis partially rediscovered sequence similarities.

This work suggests that the current TC system adopted by IUBMB is a more robust classification feature compared to substrate classification. It is quite likely that phylogenetic inference is a more sensitive indicator of homology than simple sequence similarity or identity. Thus, it appears worthwhile to test the performance of phylogeny-based methods to relate the substrate specificities of membrane transporters.

When trying to completely block the transport of a certain substrate across a particular membrane of an organism it is hard to rely only on the TC information because one substrate can be transported by several transporters from different TC families. One possible explanation in fact is that transporters assigned to different sequence families might actually share a similar 3D structure and the structural similarity might provide an indication about the evolution of the transporter function. Such studies require more sensitive search methods like AlignMe [31].

## Conclusions

We observed that classifying membrane transporters according to TC families gives more accurate results than classifying them according to substrate families. At the strictest threshold of 1e-20 for normalized E-values, predictions based on BLAST and HMMER result generally in high precision, but a huge fraction of the data remains unclassified. We suggest an acceptable threshold of 1e-8 for both programs where at least three quarters of the sequences are classified with a reasonably high accuracy. Researchers who wish to apply these thresholds in their studies should multiply these thresholds by the size of the database they search against. On the other hand, MEME showed unsatisfactory behavior for thresholds below 1e-8. Prediction of TC families split from substrate families showed satisfactory results implying that the application of substrate information as a pre-filter would improve the prediction results. The analysis and suggested thresholds in this study should be useful to those who wish to transfer transporter functional annotations across species without having to build a new phylogeny such as for the TC system. With respect to substrate annotation, the findings of this work may be combined with those of Schaadt et al. [6] who established amino acid composition for substrate annotation of transporters, and with the work of Saier MH Jr. [32].

## Methods

### Overview of the data

In the training part of this work, we used three sets of membrane transporter sequences from *Ec* (155), *Sc* (177), and *At* (158). In each case, we require that the transporter has been classified in the TC system and that TC/substrate annotations are based on experimental evidence. The sequences and annotations were retrieved from the databases TransportDB [2], SGD [3] and Aramemnon [4], respectively. From TransportDB we downloaded 354 sequences of *Ec* transporters. Among them, 157 have experimentally confirmed annotations about substrate and transporter class. *Sc* transporters were extracted from a list of 6752 ORFs downloaded from SGD. 900 transporters existed in verified ORFs among which 788 had a non-hypothetical function. Only 178 transporters had a clear TC family membership which was obtained by BLASTing SGD extracted transporters against the *Sc* TransportDB by requiring an E-value of 0.0 and a sequence identity of 100%. In Aramemnon, we used the keywords ‘transport’ and ‘carrier’ to download 616 transporter sequences from which 159 non-putatives with clear TC classification were extracted. Thereafter, we constructed subsets according to the TC system and according to substrates for later analysis. Obviously, matching a sequence correctly to a particular TC subfamily based on sequence similarity is only possible if this TC subfamily originally contains at least two members (if we take one out for testing, there is at least still one left). Thus, we considered only TC classes with more than one member. Additionally, we also downloaded functional descriptions from the Pfam database [33] for the transporters in the three organisms to assist the substrate information extracted from the individual databases. If substrate information from Pfam conflicted with the original substrate information, the Pfam information was discarded.

The transporters of the three organisms are annotated to 53 (*Ec*), 29 (*At*), and 34 (*Sc*) different TC families. Subclass 2.A (including uniporters, symporters, andantiporters) and subclass 3.A (P-P-bond-hydrolysis-driven transporters) were the most common TC subclasses. In *Sc* and *At*, the Major Facilitator Superfamily 2.A.1 accounts for nearly 40% of all transporters while in *Ec* it is the second largest family after the ATP-binding Cassette (ABC) Superfamily 3.A.1. Shared TC families belong mostly to TC classes Electrochemical Potential Driven Transporters (class 2) and the Primary Active Transporters (class 3).

For the testing part, we created four datasets of experimentally annotated human transporters (*Hs*). Sugar, amino acid, and metal transporter sets were extracted from the ChEMBL database [34]. Experimentally validated phosphate transporters were obtained from Uniprot [35]. We note that the set of metal transporters contains several proteins that transport several extra substrates besides the metal ion as well.

### Prediction tools

The statistical significance of the sequence similarity between an input sequence and sequences in the input set was determined using the well-known tools BLAST [16] and HMMER [17]. The MEME program suite [18] version 4.6.0 was used to identify enriched sequence motifs in sets of transporter sequences from one organism belonging to the same TC family or that transport the same substrate. Later, the MAST program from the MEME suite provided a score when statistically significant motifs were identified in the sequences from the other organisms. Additionally, we used the tool ggsearch36 from the FASTA suite [36] to test whether sequences transporting the same substrate express not only local but also global sequence similarity.

First, we used NCBI BLAST version 2.2.23 and HMMER version 3.0 for pairwise comparisons of all 90 *Ec* transporters against the 84 *At* transporters that belong to 14 shared TC families. In the MEME analysis, we used only common *At* and *Ec* TC families with two or more members i.e. 71 *Ec* transporters and 77 *At* transporters belonging to 7 TC families. Next, we aligned the 98 *Ec* transporters belonging to 18 TC families against 131 *Sc* transporters. *Ec* and *Sc* shared 14 TC families that could be searched by MEME involving 87 transporters from *Ec* and 127 from *Sc*. Finally, we used BLAST and HMMER to compare 157 *Sc* transporters from 23 TC families against 141 *At* transporters. *At* and *Sc* shared 12 TC families involving 130 transporters from *At* and 120 from *Sc*. Repeatedly, we used sequences from different organisms but belonging to the same TC families as inputs and test sets for the classifiers to test the quality of the prediction. For identifying enriched sequence motifs with MEME, the sequences must be grouped into families that are likely to share motifs. Here, we used MEME to determine up to 3 motifs in each shared TC family between each pair of organisms; 7 such TC families for (*At*- *Ec*), 14 for (*Sc*- *Ec*), and 12 for (*At*- *Sc*). BLAST E-values were normalized by the number of residues in the searched database (see Results section). HMMER E-values were normalized by the number of hits.

In order to identify reliability thresholds at which functional information can be safely transferred between organisms, we tested thresholds (1e-20, 1e-16, 1e-12, 1e-8 and 1e-4) for the E-values and evaluated prediction accordingly. We calculated the accuracy measures precision (positive predictive value), recall (sensitivity) and F-measure (equations 1, 2, 3) at each threshold to evaluate the prediction performance (Tables 2, 3, 4, 5, 6 and 7). Precision emphasizes the role of unexpected results whereas recall emphasizes the role of missing classification points. F-measure is a suitable accuracy measure considering precision and recall as we want precision and recall to be evenly weighted. High precision points at a strong prediction boundary while members of other classes rarely match the current class. High recall points at strong similarity within the class members as they rarely match members of other classes. For an actual TC or substrate class, a false negative is a membrane transporter from the class that is predicted to belong to another class, while a false positive is membrane transporter from another class that is predicted to belong to the current class. An example confusion matrix is illustrated in Table 10.

**Table 10:** An example confusion matrix predicting TC class 3.A.1 members

		**Predicted class**	
		**3.A.1**	**Other classes**
**Actual Class**	**3.A.1**	TP	FN
	**Other classes**	FP	TN

A confusion matrix corresponding to our method of calculating accuracy measures for the TC and substrate classifications. TPs are members of the actual class correctly classified to the same class from the other organism; Members are considered FNs if they were classified to another class. FPs are members of the other classes that were predicted to belong to the actual. TNs are members of other classes predicted to belong to other classes.

(1) $$\mathrm{Precision}=\frac{\mathrm{tp}}{\mathrm{tp}+\mathrm{fp}}$$

(2) Recall=tptp+fn

(3) $$F-\mathrm{measure}=2\times \left(\frac{\mathrm{precision}\times \mathrm{recall}}{\mathrm{precision}+\mathrm{recall}}\right)$$

## Abbreviations

Ec: Escherichia coli; Sc: Saccharomyces cerevisiae; At: Arabidopsis thaliana.

## Competing interests

The authors declare that they have no competing interests.

## Authors’ contributions

AB and VH conceived this study. AB wrote scripts, compiled data-sets, and performed the data analysis. AB and VH analyzed the data and jointly wrote the manuscript. Both authors read and approved the final manuscript.

## Supplementary Material

Additional file 1: Tables S1–S3 Considered transporters in organisms EC, AT and SC respectively. Click here for file

Additional file 2: Table S4 Complete annotation results of the pairs (*Hs*, *At*) and (*Ec*, *Hs*). Click here for file

Additional file 3: Tables S5–S6 Results of FASTA global searches. Click here for file

Additional file 4 Heatmap of BLASTing Ec substrate-TC families against At families. Click here for file

Additional file 5 Heatmap of BLASTing Ec substrate-TC families against Sc families. Click here for file
