# Supplementary figures and images for: Transferring functional annotations of membrane transporters on the basis of sequence similarity and sequence motifs
Source: BMC Bioinformatics. 2013 Nov 28;14:343. doi: 10.1186/1471-2105-14-343 (PMC4219331; doi:10.1186/1471-2105-14-343)

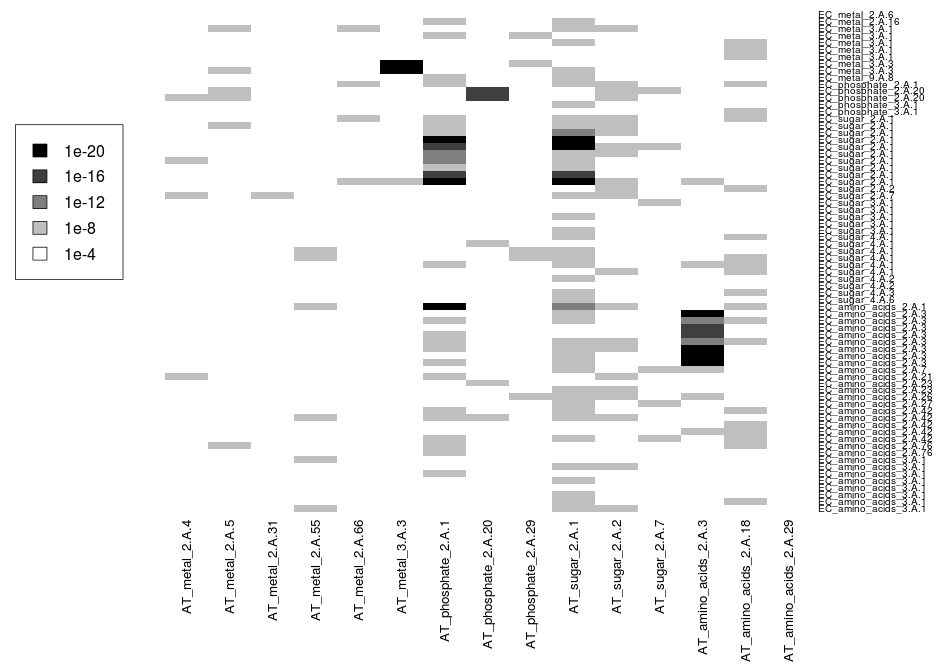

Supplement: Additional file 4 — Heatmap of BLASTing Ec substrate-TC families against At families. [file 1471-2105-14-343-S4.png]

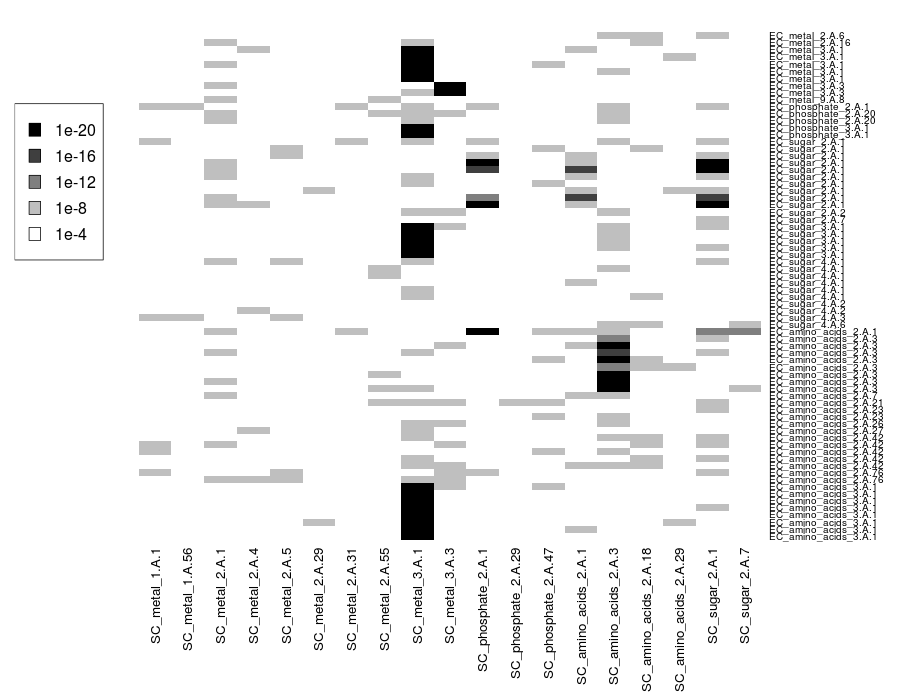

Supplement: Additional file 5 — Heatmap of BLASTing Ec substrate-TC families against Sc families. [file 1471-2105-14-343-S5.png]
